# Effectiveness of a technology-assisted, family volunteers delivered, brief, multicomponent parents’ skills training intervention for children with developmental disorders in rural Pakistan: a cluster randomized controlled trial

**DOI:** 10.1186/s13033-021-00476-w

**Published:** 2021-05-31

**Authors:** Syed Usman Hamdani, Zill-e- Huma, Nadia Suleman, Parveen Akhtar, Huma Nazir, Aqsa Masood, Mahjabeen Tariq, Ahmareen Koukab, Erica Salomone, Laura Pacione, Felicity Brown, Stephanie Shire, Siham Sikander, Chiara Servili, Duolao Wang, Fareed Aslam Minhas, Atif Rahman

**Affiliations:** 1 Institute of Psychiatry, Rawalpindi Medical University (RMU) and Benazir Bhutto Hospital, Rawalpindi, Pakistan; 2 University of Liverpool, Liverpool, UK; 3 Human Development Research Foundation, Islamabad, Pakistan; 4 Department of Mental Health and Substance Abuse, World Health Organisation, Geneva, Switzerland; 5 Department of Psychology, University of Milan-Bicocca, Milan, Italy; 6 Department of Psychiatry, Division of Child and Youth Mental Health, University of Toronto, Toronto, ON Canada; 7 Research and Development Department, War Child Holland, Amsterdam, The Netherlands; 8 Institute of Social Science Research, University of Amsterdam, Amsterdam, The Netherlands; 9 Special Education and Clinical Sciences, College of Education, University of Oregon, Eugene, OR USA; 10 Liverpool School of Tropical Medicine, Liverpool, UK

**Keywords:** Developmental disorders, WHO mhGAP-IG, Family Volunteers, Technology assisted task-shifting, Low income settings, Caregivers’ skills training, Low resource settings

## Abstract

**Background:**

Globally, there is a large documented gap between needs of families and children with developmental disorders and available services. We adapted the World Health Organization’s mental health Gap-Intervention Guidelines (mhGAP-IG) developmental disorders module into a tablet-based android application to train caregivers of children with developmental disorders. We aimed to evaluate the effectiveness of this technology-assisted, family volunteers delivered, parents’ skills training intervention to improve functioning in children with developmental disorders in a rural community of Rawalpindi, Pakistan.

**Methods:**

In a single-blinded, cluster randomized controlled trial, 30 clusters were randomised (1:1 ratio) to intervention (n = 15) or enhanced treatment as usual (ETAU) arm (n = 15). After screening, 540 children (18 participants per cluster) aged 2–12 years, with developmental disorders and their primary caregivers were recruited into the trial. Primary outcome was child’s functioning, measured by Childhood Disability Assessment Schedule for Developmental Disorders (DD-CDAS) at 6-months post-intervention. Secondary outcomes were parents’ health related quality of life, caregiver-child joint engagement, socio-emotional well-being of children, family empowerment and stigmatizing experiences. Intention-to-treat analyses were done using mixed-models adjusted for covariates and clusters.

**Results:**

At 6-months post-intervention, no statistically significant mean difference was observed on DD-CDAS between intervention and ETAU (mean [SD], 47.65 [26.94] vs. 48.72 [28.37], Adjusted Mean Difference (AMD), − 2.63; 95% CI − 6.50 to 1.24). However, parents in the intervention arm, compared to ETAU reported improved health related quality of life (mean [SD] 65.56 [23.25] vs. 62.17 [22.63], AMD 5.28; 95% CI 0.44 to 10.11). The results were non-significant for other secondary outcomes.

**Conclusions:**

In the relatively short intervention period of 6 months, no improvement in child functioning was observed; but, there were significant improvements in caregivers’ health related quality of life. Further trials with a longer follow-up are recommended to evaluate the impact of intervention.

*Trial registration* Clinicaltrials.gov, NCT02792894. Registered April 4, 2016, https://clinicaltrials.gov/ct2/show/NCT02792894

## Background

Developmental disorders are lifelong conditions characterized by early childhood onset and a delay in central nervous system development and maturation. They include conditions such as Autism Spectrum Disorder (ASD) and Intellectual Disabilities (ID) [1]. Developmental disorders cause substantial economic and social burden on families and societies globally [2, 3]; therefore, addressing developmental disorders is a priority for the global health agenda [4]. The World Health Organization (WHO) has developed evidence-based guidelines, published in the Mental Health Gap Intervention Guide (mhGAP-IG) for the management of priority mental health conditions in low resource settings globally [5]. The WHO mhGAP-IG recommends caregivers’ skills training for the management of children with developmental disorders in low-resource settings. The WHO mhGAP guidelines take a trans-diagnostic approach (a diagnosis is not required for participation in the program) and are designed to be delivered by non-specialists (health workers, primary health care physicians, nurses). These guidelines are recommended to be integrated into existing community services [6].

In Pakistan, over 6% of children suffer from a developmental disorder (ID or ASD) and like other low resource settings, most children and families receive no intervention or care. Specialist services are rare, concentrated in urban areas and inaccessible to the majority. There is considerable stigma and discrimination affecting children with developmental disorders and their families. Thus, the treatment gap for developmental disorders in rural Pakistan is almost 100% [7, 8]. The Ministry of Health in Pakistan is implementing the WHO mhGAP in primary health care settings to bridge the treatment gap for priority mental health conditions including childhood developmental disorders in Pakistan. Like many other low resource settings that have neither the trained human resource nor the mechanism to ensure program fidelity at-scale in real world settings, there are many contextual challenges to mhGAP implementation in Pakistan [9].

In recent years, use of mobile and internet technology has been recommended to address the barriers to access and improve quality of mental health care in low resource settings [10]. As a part of mhGAP implementation in a pilot sub-district of Rawalpindi, Pakistan, we converted the WHO mhGAP guidelines into training videos for caregivers of children with developmental disorders and hosted them on a tablet device. Family Volunteers (FVs) were trained to deliver this training to the caregivers of children with developmental disorders [7]. The training videos were interactive, allowing family volunteers and members to discuss each scenario in the context of their own lives and develop individualised management plans for their children. To pilot test the intervention, we identified and trained 10 family volunteers in implementing technology assisted, evidence-based, WHO mhGAP guidelines with 70 families and children with developmental disorders. The program was found to be feasible, acceptable and resulted in change in child’s outcomes [7].

The aim of present study was to evaluate the effectiveness of a scaled-up model of technology assisted, family volunteers delivered, brief, multicomponent parents’ skills training intervention for the management of developmental disorders in a low resource setting of rural Pakistan [11].

## Methods

### Study design

The effectiveness of intervention was evaluated in a two arm, single-blinded cluster randomized controlled trial (cRCT). As the intervention was delivered by ‘Family Volunteers’ in village-based groups, cluster randomized controlled design was considered appropriate to avoid contamination between the intervention and control arms. Primary outcome (child functioning) was evaluated at 6-months post-intervention. Implementation effectiveness was evaluated using mixed-methods research which has been described elsewhere (*forthcoming publication*).

### Study settings and participants

The study was conducted in a rural sub-district of Gujar Khan in Rawalpindi district, Pakistan. Gujar Khan sub-district is a typical rural setting in northern Punjab and Pothohar region, situated about 35 km south east of the Rawalpindi city and has a population of about 1 million. The sub-district is divided into 30 rural Union Councils (UCs). Each UC is the smallest administrative unit within a district and it formed our unit for cluster randomisation. The trial participants were children (a) aged between 2 and 12 years, residing in the study sub-district for the duration of the study. A two-stage screening process included (a) positive score on any of the Ten Questions Screen questionnaire items # 1, 4, 5, 7, 8, 9, 10 for neurodevelopmental delay [12]—a cross culturally valid instrument to screen children with developmental difficulties [13]; and (b) clinical assessment of screened-positive children with developmental delays and disorder(s) according to the WHO mhGAP developmental disorders guidelines for clinical assessment in primary healthcare settings by a trained clinical psychologist. The ethics approval for this study was obtained from the Institutional Review Board and Ethics Board of the Rawalpindi Medical University and Allied Hospitals Rawalpindi (ethical approval certificate number ERC/RMU/29/01/2017) and Human Development Research Foundation, Islamabad (IRB/002/2016), Pakistan. All participants provided written informed consent before participating in the study. The trial protocol has been published [11].

### Randomization and blinding

The unit of randomization was a Union Council (UC—the smallest administrative unit in rural sub-district). Thirty UCs were randomized to intervention and control arms on a 1:1 allocation ratio using a permuted-block randomization method with the block size of 6. Allocation of clusters was carried out by an independent researcher not involved in the conduct of the trial before the recruitment of participants in the study. The outcome assessment team, trial statistician and principal investigator were blind to the allocation status of trial participants. The fidelity of the masking was assessed by asking the assessors to guess the allocation status of each trial participants after post-assessment.

### Intervention

We developed the intervention based on the guidelines of WHO mhGAP-IG module on developmental disorders. The adapted intervention consists of nine sessions delivered in group format to caregivers of children with developmental disorders. The programme aims to provide evidence-based strategies to caregivers of children with developmental disorders that can be implemented in low resource settings. The strategies target to promote child’s communication, socioemotional development and adaptive behaviours; manage co-morbid conditions in children; provide practical guidance on managing motor difficulties in children and coping skills to improve psychological well-being of caregivers. The intervention was delivered to caregivers of children with developmental disorders in 9 sessions. The nine intervention sessions are briefly described in Table 1 (*Intervention manual and online training resources are freely available and can be accessed here* https://fansforkids.org/).

**Table 1:** Outline of intervention sessions and content

Sessions	Content	Intervention density
Session 1: Getting to know developmental disorders	Introducing program and engaging caregivers and family Psycho-educating caregivers about child development and developmental delays and disorders Strategies for caregivers to engage children and promote child development Highlighting the role of family, school and community in helping and supporting children with special needs	A 90 min session in week-1
Session 2: Causes and prevention of developmental disorders	Psychoeducation about causes and prevention of developmental disorders/delays before, during and after child birth and in early childhood	A 90 min session in week-2
Session 3: Learning through play and learning to play	Strategies to manage caregiver’s stress Play based activities to promote caregiver-child interaction and child’s learning	A 90 min session in week-3
Session 4: Understanding and promoting communication	Psycho-educating caregivers about understanding child’s communication (by observing child’s words, gesture and challenging behaviour) Skills based strategies for caregiver to promote child’s communication ○ Respond to child’s every communication by showing a gesture and talking at a level that he/she can understand ○ Look and listen to notice when a child wants something and wait for a few seconds to see if he/she will ask for ○ Help the child to know how to ask for something with a gesture and words	A 90 min session in week-4
Session 5: Managing challenging behaviours	Skills based strategies for caregivers to manage child’s challenging behaviours ○ Managing child’s challenging behaviour by looking and listening for signals before the challenging behaviour ○ Responding to child’s skills and appropriate behaviour with praise and encouragement ○ Responding to challenging behaviour by teaching the child words and gestures to ask Use of picture schedules to help the child understand routines and stay regulated	A 90 min session in week-5
Session 6: Managing everyday life situations	Psycho-educating caregivers that children can learn new skills for everyday life by practicing with caregiver’s help and assistance Skills based strategy (i.e. break and teach) and four levels of help for caregivers to teach child every day skills	A 90 min session in week-6
Session 7: Managing caregiver’s stress through problem solving and group support	Strategies to manage caregiver’s stress ○ Stress management through slow breathing ○ Problem solving ○ Ensuring family support ○ Mobilizing group support to manage practical problems	A 90 min session in week-7
Session 8: Strategies to manage comorbid conditions in children with developmental disorders/delays	Psychoeducation and skill based strategies to manage co-morbid conditions including epilepsy, adolescent depression and Attention Deficit Hyperactivity Disorder (ADHD) Promoting community based rehabilitation, addressing stigma and promoting human rights according to the WHO mhGAP-IG guidelines	A 90 min session in week-8
Session 9: Strategies to manage motor difficulties in children with developmental disorders/delays	Practical guidance on managing motor difficulties in children with developmental disorders	A 90 min session in week-9

**Fig. 1:**
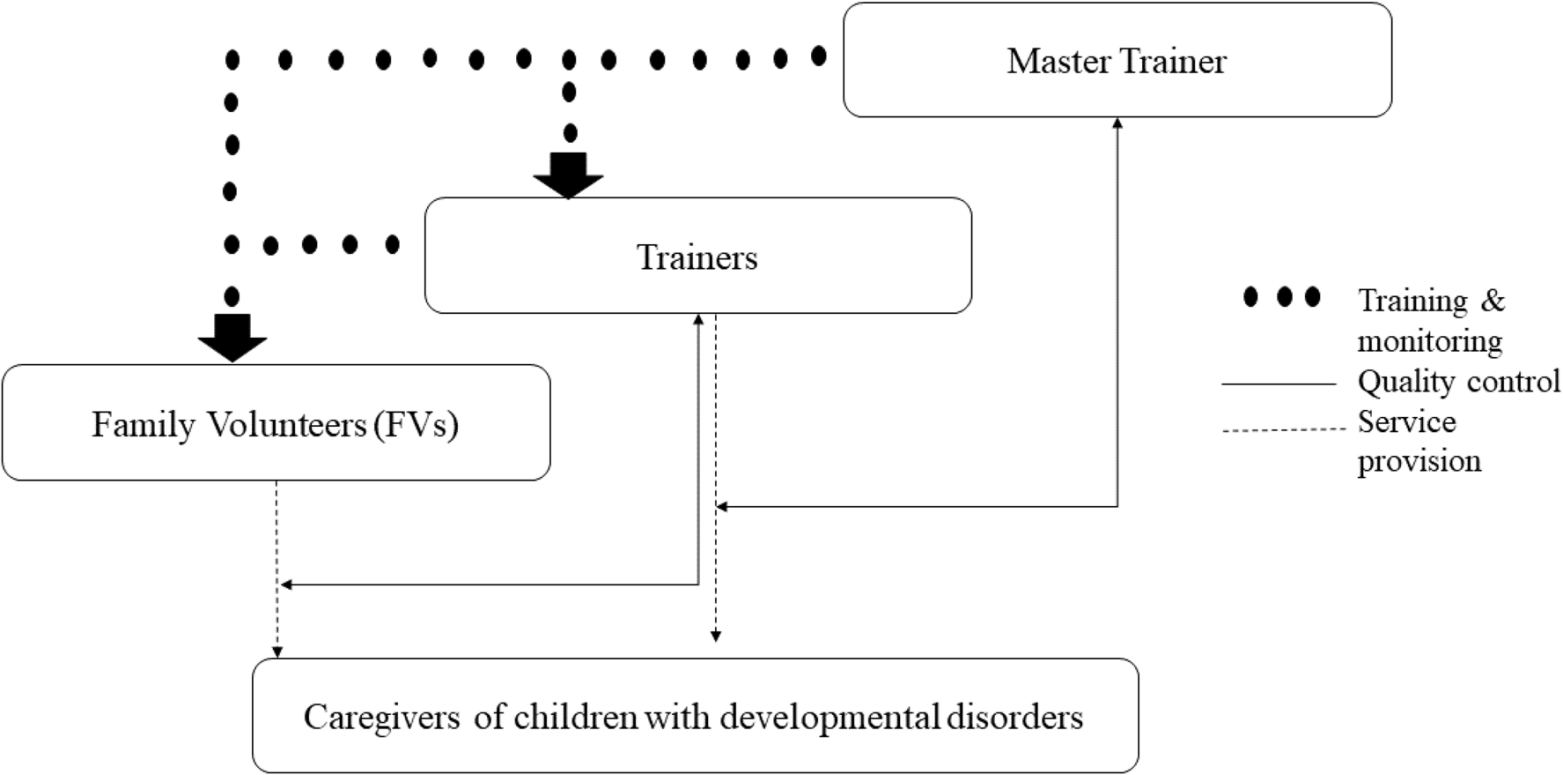
Master trainer (UH); trainers (had at-least 16 years of education and 1 year of experience in working with children and families with developmental disorders; Family Volunteers (FVs) (parents or caregivers of children with developmental disorders, had at least eight grades of formal education, are voluntarily willing to be trained and supervised by the trainers for at-least 6-months duration of the programme and cascade the training to 4–5 families in their villages) (Adapted from Murray et al. [14])

In the adaptation of the intervention from the WHO mhGAP-IG, we employed several strategies to aid scale-up of intervention in under-resourced settings; *firstly*, based on the formative research [7] and in consultation with mental health experts and program trainers, the content of the intervention modules was simplified and contextualised to make the program content relatable for the parents with limited literacy skills. *Secondly*, an android application consisting of simple training videos, hosted on a tablet device was used to train parents, taking out the need for specialist to deliver the training. The intervention guidelines were broken down into training scenarios which were converted into narrative scripts by a panel of experts. The training scenarios incorporated all core techniques and principles of intervention. The key psycho-educational contents (‘key messages’ and strategies) of caregivers’ skills training were incorporated into ‘real-life’ narratives of the lives of three children with developmental disorders, their family members, and other supporting characters. Culturally appropriate real-life characters for the Family Volunteers (FVs) were developed. An artist converted the characters into ‘Avatars’. The training scenarios were shared with the group of parents and community health workers (not involved in the study). Their feedback was incorporated to refine the narratives. The narratives were interactive, with pause buttons and instructions; allowing family members to discuss each scenario in the context of their own lives; develop individualized management plans for their child based on the information provided; practice skills through role plays; discuss ways of increasing participation in communal life; and share problem-solving strategies. *Thirdly*, we used cascade model for the training and supervision of FVs and parents [14] (Fig. 1). First, the master trainer trained 10 trainers. The training of trainers was conducted using the android application hosted on a tablet device. The training consisted of 10-days class-room training followed by case studies under the supervision of master trainer. The master trainer performed live competency rating of the trainers on the case studies using an adapted version of ENhancing Assessment of Common Therapeutic factors (ENACT) for caregiver skills training for developmental disorders [15]. The trainers had completed at-least a Masters in Clinical Psychology and had at-least 1 year of experience in working with children and families with developmental disorders. Only competent trainers (mean score above 2.5 on all domains of adapted version of ENACT) were allowed to train and supervise the Family Volunteers (FVs). Each trainer trained FVs in 9 weekly group sessions followed by competency rating using adapted version of ENACT. The trainers cascaded down the training to 62 FVs using tablet-based tool, who, then, delivered the programme to the 4–5 families in their villages. Each trainer was assigned a case load of 4–6 FVs for training and supervision. These FVs were either the parents or were related to the children with developmental disorders, had at-least eight grades of formal education, and volunteered to be trained and supervised by the trainers for at-least 6-months duration of the programme.

The FVs were fortnightly supervised by the trainers during the program delivery; similarly, trainers were supervised fortnightly by the master trainer. The fidelity of program delivery by the FVs was assessed by the trainers. To assess the fidelity, the trainers randomly selected and rated 20% of the sessions delivered by the FVs using adapted ENACT.

The intervention was delivered to caregivers of children with developmental disorders by the FVs using the tablet-based android application in weekly group sessions, for 9-weeks. To deliver intervention sessions, a tablet device (*an intervention delivery aid*) was provided to each FV for the duration of the program. The participation of caregivers in trainings sessions was tailored to the individual needs of their child. After 9-weeks of program implementation, FVs provided supervision to the caregivers in implementing the intervention strategies with their children in fortnightly group sessions with caregivers, delivered over a period of 4-months. All group sessions were organized at the local primary healthcare facility. No financial remuneration was provided to FVs for the program delivery.

### Enhanced Treatment As Usual (ETAU)

Evidence-based mental health care is currently not available in primary healthcare centres. Treatment as usual for childhood developmental disorders in primary healthcare centres of Rawalpindi consists of a range of complementary/alternative treatment regimes, such as multi-vitamin syrups and tablets, prescribed by the primary health care physicians. For this study, the treatment as usual was enhanced in two ways: (a) Community Health Workers (CHWs), in the ETAU arm received training in recognising signs and symptoms of developmental disorders and making referrals to their primary care physicians for treatment; and (b) the primary care physicians received a half day orientation session on identification, management and referral guidelines of WHO mhGAP on developmental disorders by the specialists at Institute of Psychiatry, WHO Collaborating Centre for mental health in Pakistan. Data on health services accessed by trial participants in both arms is presented in Table 2.

**Table 2:** Health services utilization across two arms at baseline and during past 6-months (N = 540)

	Group	Baseline			6-months post-intervention		
		n (%)	Mean number of visits (SD)	Mean duration in minutes (SD)	n (%)	Mean number of visits (SD)	Mean duration in minutes (SD)
Education services							
Main stream school	ETAU arm	65	5.88 (0.57)	5.85 (0.66)	81	5.90 (0.30)	5.89 (0.89)
	Intervention arm	54	5.85 (0.40)	5.72 (0.71)	79	5.87 (0.43)	5.77 (1.04)
Outpatient services							
Traditional healer	ETAU arm	2	3.00 (1.41)	7.50 (3.53)	9	2.44 (1.59)	16.67 (11.45)
	Intervention arm	2	2.00 (0)	7.50 (3.53)	9	4.56 (6.63)	12.78 (7.12)
Mental health professional	ETAU arm	0	0	0	0	0	0
	Intervention arm	0	0	0	0	0	0
Medical doctor	ETAU arm	5	16.60 (13.53)	28 (4.47)	7	54 (78.4)	60.71 (43.05)
	Intervention arm	10	11.90 (16.42)	42 (31.46)	7	2.57 (1.71)	62.14 (53.68)
Community health worker	ETAU arm	13	3.38 (3.66)	10.38 (8.47)	33	2.82 (2.28)	10.09 (7.32)
	Intervention arm	10	5.40 (5.48)	15.50 (11.41)	13	4.85 (6.65)	9.54 (5.72)
Any others services	ETAU arm	5	3.40 (1.94)	11.00 (11.93)	0	0	0
	Intervention arm	5	5.80 (4.49)	11.00 (4.18)	0	0	0
Religious help and retreats	ETAU arm	ETAU arm	4.04 (15.52)	–^a^	89	5.51 (5.92)	0
	Intervention arm	Intervention arm	6.07 (18.50)	–^a^	69	6.53 (7.94)	0

*ETAU* enhanced treatment-as-usual

^a^Data was missing

### Outcomes

Baseline and end point assessments (6 months’ post-intervention) on the sample of 540 caregiver–child dyads were conducted by the trained research assessment team blind to the allocation status of the trial participants.

### Primary outcome

Developmental Disorders-Children Disability Assessment Schedule (DD-CDAS) [16]: was adapted from the WHODAS-Child [17]. The DD-CDAS is a 36-item questionnaire measuring functioning and disability. The 36 items are rated on a five-point Likert scale (1 = *none* to 5 = *extreme/cannot do*). The items represent cognition, mobility, self-care, getting along with others, life activities, and participation in the society. The raw scores are sum across all the items of each domain and all 36 items for a tool. Domain and total raw scores are transformed into a range from 0 to 100. A global disability score is computed from all 36 items, where higher score indicates greater disability or difficulty in functioning. The interviewer-administered proxy version (caregiver report) of the tool because children with developmental disorders were not expected to be able to self-report. The tool has been validated for children with developmental disorders in Pakistan (DD-CDAS) by our group [16].

### Secondary outcomes

Parental health related quality of life was measured by Paediatric Quality of Life (Peds-QL) [18] family impact module. The Peds-QL is a 36-item scale comprising 8 dimensions (physical functioning, emotional functioning, social functioning, cognitive functioning, communication, worry, daily activities and family relationships) measuring parent self-reported functioning. Items are rated on a 5-point Likert scale (0 = *never* to 4 = *almost always*). Peds-QL has shown sound psychometric properties in different cultures [19, 20].

Caregiver–child interaction [21]: we proposed to video tape a 15-min caregiver–child interaction at baseline and at-endpoint for families in both arms of the study. Caregivers were asked to try home routines involving the child (e.g. feeding the child, performing domestic chores) or play based routines (e.g. playing with toys or reading a book) with their child. Caregiver’s facilitators and interrupters (including child’s engagement and distress during social communication) and joint engagement was rated. The CCI videos were singly coded by trained assessors.

Socio-emotional well-being of children was measured using Strengths and Difficulties Questionnaire (SDQ) [22]. SDQ is a parent-rated, 25 item scale distributed over five domains: emotional symptoms; conduct problems; hyperactivity/inattention; peer relationship problems, and prosocial behaviour. Each item is rated on a 3-point Likert scale (0 = *not true*, 1 = *somewhat true*, 2 = *certainly true*). A total difficulty score is calculated by adding the scores of all domain except prosocial behaviour items [22]. SDQ has been validated in Pakistani and has shown good psychometric properties [23, 24].

Family empowerment was measured by Family Empowerment Scale (FES) [25]. FES is a parent-rated, 34 items scale consisting of three subscales (family subscale [12 items], service system subscale [12 items] and community subscale [10 items]). Each item is rated on a 5-point Likert scale (1 = *not true at all* to 5 = *very true*). Scores are summed across all items for each subscale with higher scores indicating relatively more empowerment. FES has shown good internal consistency, test–retest reliability and factor structure.

Caregivers’ stigmatizing experiences were measured using Inventory of Stigmatizing Experiences (ISE) [26]—family version. ISE is a 7 items interview based measure of the extent of stigma faced by family. Each item is rated on a 5-point liker scale (1 = *never* to 5 = *always*). The responses are recoded into a binary variable with 1 reflecting presence of stigma and 0 reflecting absence of stigma. Scores are summed across all items with a maximum score of 7, with lower scores indicating relatively less stigma. The scale indicated good internal consistency.

Health services utilisation by the trial participants was measured using the Client Service Receipt Inventory [27], that has been adapted for use in children and families with developmental disorders.

The data on severe adverse events, including death of the participant due to any cause, suicide attempt and hospital admission was collected from all study participants at 6-months post-intervention follow-up.

### Statistical analysis

The data was analysed using SAS 9.3 on intention-to-treat basis. The previous studies of non-specialist delivered psychosocial interventions for children with intellectual disability and autism spectrum disorders used the effect sizes between 0.1 and 1 for the intervention outcome [6]. We aimed for a conservative effect-size estimate of 0.35 for the primary outcome measure. We proposed to recruit a sample of 540 parent–child dyads in 30 clusters (an average of 18 parent–child dyads per cluster), equally distributed between the intervention and control arms. This gave 93% power at 5% two-sided significance level with an intra-cluster correlation coefficient (ICC) of 0.01, after accounting for 15% attrition rate. To estimate the treatment effect, a linear mixed model was employed for the primary endpoint analysis, which had treatment as fixed effect, and baseline measurement of primary endpoint as covariate, and cluster as random effect. The mean difference between two treatment arms at 6-months together with its 95% confidence interval was derived from the mixed model. Covariate-adjusted mixed model of primary endpoint was also performed by adding pre-specified covariates (age, gender and baseline severity) at baseline into the above model. Subgroup analysis was also performed for the pre-specified covariates (age, gender and baseline severity), used in the covariate adjusted analysis. Adjusted and subgroup analysis were based on covariates at baseline without missing values. Sensitivity analysis was performed on the covariates with missing values imputed [28]. Missing data was treated as missing at random in the mixed model analysis and no imputation of primary and secondary endpoints was made. All trial analyses were described in detail in the finalised and signed statistical analysis plan before unmasking the data and have been described in the published trial protocol.

### Procedures

The current trial to evaluate the effectiveness of mhGAP-IG based caregivers’ skills training was embedded within the implementation of WHO mhGAP program in Pakistan. 540 parent–child dyads, who fulfilled the eligibility criteria for participation in the trial, from 30 UCs (on an average 18 parent–child dyad in each UC) were enrolled in the study between February and April, 2017. All primary caregivers of the children provided written informed consent for participation in the study. The details of recruitment and trial procedures can be accessed from the registered and published trial protocols [11].

## Results

**Fig. 2:**
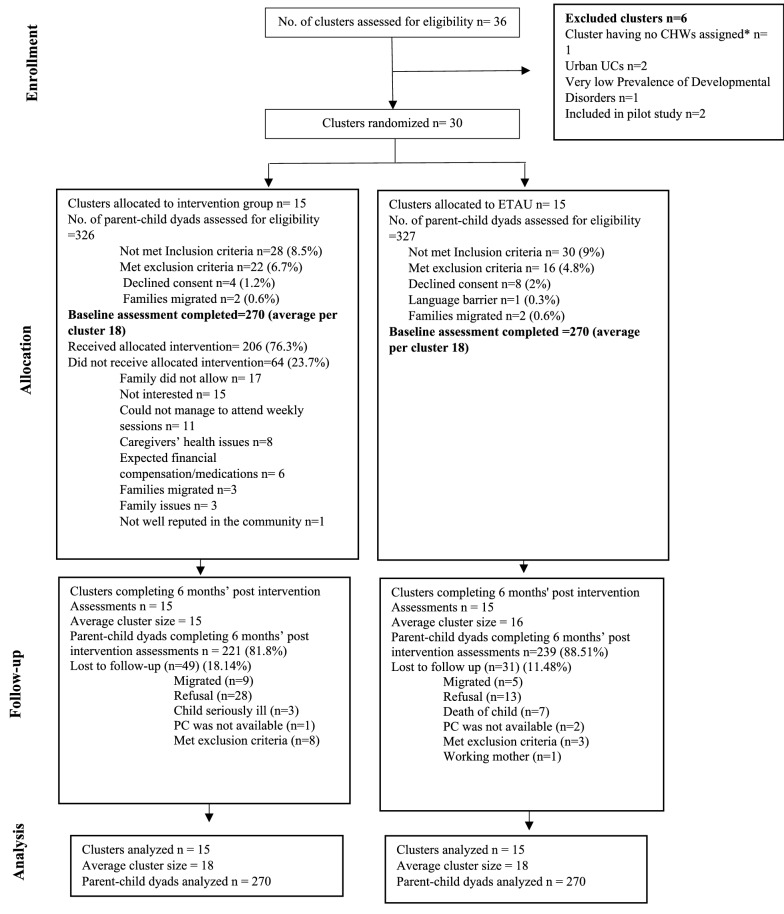
Flow of participants through trial

The findings of the trial are reported following the recommendations of the Consolidated Standards of Reporting Trials (CONSORT) 2010 statement: extension to cluster randomized trials [29]. Figure 2 depicts participant flow through the trial. In 30 eligible UC clusters, 653 parent-child dyads were assessed for eligibility and 540 parent–child dyads who met the eligibility criteria were enrolled in the trial (average cluster size 18 participants). We were able to collect primary endpoint data at 6 months’ post-intervention from 460/540 (85.18%) trial participants.

The mean (SD) age of children was 6.72 (± 2.76) years and 60% of the children were males (320/540). Based upon the clinical assessment by psychologists 44% (239/540) of the children had cognitive difficulties, 40% (216/540) of children had cognitive difficulties with motor difficulties, and 5.9% (32/540), 2.6% (14/540) and 7.2% (39/540) had motor difficulties, communication difficulties and Down Syndrome, respectively. Only 27.4% (148/540) of children were attending school. Mothers were primary caregivers in 96% (520/540) of cases. Regarding mothers’ literacy, 33% (177/540) of mothers had no formal education; whereas, 56% (303/540) of mothers had completed some schooling (7 [± 1.5] years of schooling). In 74% (399/540) of families, caregivers had consanguineous marriages and 35% (187/540) of the children had positive family history for developmental disorders. There was no significant difference between the intervention and control arms in demographic characteristics (Table 3).

**Table 3:** Demographic characteristics (N = 540)

Characteristic	Total (N = 540)	Intervention arm (n = 270)	ETAU arm (n = 270)
Age of child in years	6.72 (2.76)	6.59 (2.66)	6.85 (2.86)
Age in categories			
2–8 years	378 (70.0%)	195 (72.2%)	183(67.8%)
9–12 years	162 (30.0%)	75 (27.8%)	87 (32.2%)
Sex of child			
Male	320 (59.3%)	159 (58.9%)	161 (59.6%)
Female	220 (40.7%)	111 (41.1%)	109 (40.4%)
Caregivers’ relationship with child			
Mother	520 (96.3%)	259 (95.9%)	261 (96.7%)
Sister	4 (0.7%)	2 (0.7%)	2 (0.7%)
Paternal aunt	6 (1.1%)	4 (1.5%)	2 (0.7%)
Maternal aunt	3 (0.6%)	1 (0.4%)	2 (0.7%)
Grandmother	7 (1.3%)	4 (1.5%)	3 (1.1%)
Type of developmental difficulties			
Cognitive difficulties	239 (44.3%)	116 (43.0%)	123 (45.6%)
Motor difficulties	32 (5.9%)	15 (5.6%)	17 (6.3%)
Cognitive difficulties with motor difficulties	216 (40.0%)	108 (40.0%)	108 (40.0%)
Communication difficulties	14 (2.6%)	8 (3.0%)	6 (2.2%)
Down syndrome	39 (7.2%)	23 (8.5%)	16 (5.9%)
Mother’s education			
No schooling	177 (32.8%)	84 (31.1%)	93 (34.4%)
Primary (6 years)	126 (23.3%)	56 (20.7%)	70 (25.9%)
Middle (8 years)	70 (13.0%)	40 (14.8%)	30 (11.1%)
Matric (10 years)	107 (19.8%)	52 (19.3%)	55 (20.4%)
Intermediate (12 years)	28 (5.2%)	16 (5.9%)	12 (4.4%)
College and university (16 years)	32 (5.9%)	22 (8.1%)	10 (3.7%)
Father’s education			
No schooling	76 (14.1%)	42 (15.6%)	34 (12.6%)
Primary (6 years)	71 (13.1%)	28 (10.4%)	43 (15.9%)
Middle (8 years)	115 (21.3%)	60 (22.2%)	55 (20.4%)
Matric (10 years)	198 (36.7%)	99 (36.7%)	99 (36.7%)
Intermediate (12 years)	43 (8.0%)	25 (9.3%)	18 (6.7%)
College and university (16 years)	27 (5%)	13 (4.8)	14 (5.1)
Not applicable	10 (1.9%)	3 (1.1%)	7 (2.6%)
Total monthly income	540	270	270
Mean (SD)^a^	19,236.65 (17,617.78)	19,543.58 (19,194.36)	18,929.71 (15,915.82)

^a^Amount in Pakistani rupees

At baseline, the DD-CDAS total score in the intervention and control arms were (mean [SD] 56.13 [22.56] vs. 53.65 [23.08]). At 6-months post-intervention, although, the intervention arm did show more improvement in disability scores on DD-CDAS from baseline as compared to control arm, the difference on DD-CDAS total score between intervention and control arm at endpoint was not statistically significant (mean [SD], 47.65 [26.94] vs. 48.72 [28.37]), AMD, − 2.63; 95% CI − 6.50 to 1.24) (Table 4).

**Table 4:** Summary of mixed model analysis of primary and secondary outcomes (N = 540)

Measurements	Visit	Descriptive statistics				Mixed model analysis	
		Intervention arm		ETAU arm		Difference in mean (95% CI)	*p*-value
		*n*	*Mean (SD)*	*n*	*Mean (SD)*		
DD-CDAS total disability score	Baseline	270	56.13 (22.56)	270	53.65 (23.08)		
	6 months post-intervention	221	47.65 (26.94)	239	48.72 (26.37)	− 2.63 (− 6.50, 1.24)	0.1820
DD-CDAS—understanding and communication	Baseline	270	59.15 (25.07)	270	58.81 (24.16)		
	6 months post-intervention	221	49.21 (26.97)	239	51.08 (27.87)	− 1.28 (− 6.53, 3.96)	0.6304
DD-CDAS—getting around	Baseline	270	45.17 (42.32)	270	43.56 (40.99)		
	6 months post-intervention	221	37.40 (43.10)	239	39.64 (41.14)	− 2.66 (− 6.75, 1.44)	0.2024
DD-CDAS—self-care	Baseline	270	65.97 (28.24)		61.02 (29.28)		
	6 months post-intervention	221	54.58 (33.46)	239	53.09 (32.77)	− 2.66 (− 6.74, 1.43)	0.2020
DD-CDAS—getting along with people	Baseline	270	51.52 (28.85)	270	49.11 (28.12)		
	6 months post-intervention	221	42.85 (30.23)	239	46.07 (30.49)	− 3.60 (− 9.63, 2.42)	0.2404
DD-CDAS—participation in society	Baseline	270	42.74 (21.84)	270	39.27 (23.30)		
	6 months post-intervention	221	37.99 (24.18)	239	38.06 (22.91)	− 1.70 (− 7.82, 4.42)	0.5862
DD-CDAS—life activities	Baseline	270	72.21 (24.76)	270	70.11 (23.99)		
	6 months post-intervention	221	63.90 (31.47)	239	64.40 (30.16)	− 1.83 (− 6.33, 2.67)	0.4245
PedsQL total score	Baseline	270	58.56 (17.37)	270	60.66 (18.69)		
	6 months post-intervention	221	65.56 (23.25)	237	62.17 (22.63)	5.28 (0.44,10.11)	0.0325
PedsQL—parental health related quality of life	Baseline	270	57.09 (20.37)	270	57.90 (22.14)		
	6 months post-intervention	221	63.92 (24.70)	237	60.58 (24.75)	4.43 (− 0.53, 9.39)	0.0797
PedsQL—family functioning	Baseline	270	71.63 (22.64)	270	74.16 (20.26)		
	6 months post-intervention	221	72.68 (24.32)	237	70.27 (25.04)	3.88 (− 1.72, 9.48)	0.1738
PedsQL—physical functioning	Baseline	270	48.61 (23.46)	270	51.74 (25.14)		
	6 months post-intervention	221	61.39 (28.93)	237	57.17 (29.46)	5.73 (− 0.53, 12.00)	0.0728
PedsQL—emotional functioning	Baseline	270	51.91 (26.79)	270	51.97 (27.17)		
	6 months post-intervention	221	61.76 (26.12)	237	56.08 (28.03)	6.23 (0.52, 11.94)	0.0325
PedsQL—social functioning	Baseline	270	65.93 (26.55)	270	65.46 (26.86)		
	6 months post-intervention	221	67.79 (30.62)	237	64.87 (30.18)	3.10 (− 3.43, 9.63)	0.3515
PedsQL—cognitive functioning	Baseline	270	65.37 (27.28)	270	65.13 (27.64)		
	6 months post-intervention	221	66.04 (29.25)	237	65.74 (29.88)	1.24 (− 4.15, 6.63)	0.6509
PedsQ—communication total	Baseline	270	66.44 (24.12)	270	71.45 (23.48)		
	6 months post-intervention	221	70.72 (28.37)	237	66.75 (28.20)	5.85 (− 0.25, 11.95)	0.0602
PedsQL—worry	Baseline	270	38.71 (22.16)	270	43.60 (22.33)		
	6 months post-intervention	221	57.58 (28.66)	237	52.85 (30.09)	6.66 (0.00,13.33)	0.0499
PedsQL—daily activities	Baseline	270	54.81 (31.15)	270	55.80 (30.95)		
	6 months post-intervention	221	57.96 (33.65)	237	56.01 (35.92)	2.95 (− 5.61, 11.52)	0.4984
PedsQL—family relationship	Baseline	270	81.72 (23.57)	270	85.17 (20.92)		
	6 months post-intervention	221	81.52 (24.35)	237	78.84 (26.59)	4.04 (− 1.65, 9.73)	0.1632
SDQ total socio emotional difficulties	Baseline	270	16.50 (5.56)	270	16.50 (6.04)		
	6 months post-intervention	221	16.24 (6.13)	238	16.98 (6.38)	− 0.79 (− 2.24, 0.66)	0.2840
SDQ—emotional symptoms	Baseline	270	3.73 (2.44)	270	3.94 (2.48)		
	6 months post-intervention	221	3.64 (2.41)	238	4.04 (2.53)	− 0.36 (− 0.97, 0.25)	0.2460
SDQ—conduct problems	Baseline	270	3.28 (1.99)	270	3.23 (2.11)		
	6 months post-intervention	221	3.49 (1.86)	238	3.61 (1.86)	− 0.13 (− 0.57, 0.30)	0.5434
SDQ—hyperactivity symptoms	Baseline	270	5.22 (2.29)	270	5.29 (2.25)		
	6 months post-intervention		4.77 (2.42)		4.90 (2.36)	− 0.12 (− 0.54, 0.31)	0.5965
SDQ—peer problems	Baseline		4.27 (2.02)		4.04 (2.21)		
	6 months post-intervention	221	4.33 (2.03)	238	4.43 (2.29)	− 0.19 (− 0.69, 0.31)	0.4550
SDQ—pro social	Baseline	270	4.49 (2.94)	270	4.96 (3.26)		
	6 months post-intervention	221	4.86 (3.09)	238	5.20 (3.06)	− 0.12 (− 0.60, 0.37)	0.6395
FES total score	Baseline	270	40.77 (11.07)	270	39.74 (10.16)		
	6 months post-intervention	221	41.41 (9.78)	237	40.89 (9.52)	0.47 (− 1.70, 2.64)	0.6701
FES—service system	Baseline	270	34.60 (12.37)	270	34.24 (10.97)		
	6 months post-intervention	221	39.24 (11.59)	237	38.72 (11.79)	0.55 (− 2.64, 3.75)	0.7344
FES—community involvement	Baseline	270	19.30 (7.48)	270	18.87 (7.08)		
	6 months post-intervention	221	20.28 (8.21)	237	19.48 (7.09)	1.117 (− 2.20, 0.607)	0.26
FES—attitude	Baseline	270	31.70 (9.31)	270	30.86 (8.60)		
	6 months post-intervention	221	32.84 (8.73)	237	32.23 (8.21)	0.53 (− 0.99, 2.05)	0.4936
FES—knowledge	Baseline	270	32.61 (10.24)	270	32.32 (9.15)		
	6 months post-intervention	221	36.14 (10.73)	237	35.45 (9.91)	0.81 (− 1.63, 3.24)	0.5160
FES—behaviours	Baseline	270	30.36 (9.76)	270	29.66 (8.75)		
	6 months post-intervention	221	31.95 (8.78)	237	31.42 (8.45)	0.46 (− 1.72, 2.65)	0.6768
ISE	Baseline	270	2.46 (2.03)	270	2.51 (2.27)		
	6 months post-intervention	221	2.23 (2.05)	237	2.42 (2.23)	− 0.21 (− 0.70, 0.28)	0.3967

*ETAU* enhanced treatment-as-usual, *DD-CDAS* Developmental Disorders-Children Disability Assessment Schedule, *PedsQL* Paediatric Quality of Life Inventor—family impact module, *SDQ* Strengths and Difficulties Questionnaire, *FES* Family Assessment Schedule, *ISE* Inventory of Stigmatizing Experiences

At 6-months post-intervention, there was statistically significant improvement in parental health related quality of life (PedsQL) total score (mean [SD] 65.56 [23.25] vs. 62.17 [22.63], AMD 5.28; 95% CI 0.44 to 10.11) and individual domain scores on; emotional wellbeing, (mean [SD] 61.76 [26.12] vs. 56.08 [28.03]), AMD 6.23; 95% CI 0.52 to 11.94) and worry domain (mean [SD] 57.58 [28.66] vs. 52.85 [30.09], AMD 6.66; 95% CI 0.00 to 13.33). The results of covariate adjusted analysis show that baseline severity, age and gender of the child did not influence the intervention’s effects on caregiver quality of life (Table 5). Since, the study was conducted in a conservative rural community settings of Pakistan, only 10% of the study sample consented to be video recorded for caregiver–child interaction. The intervention did not result in improving caregiver-child interaction, socio-emotional difficulties and family empowerment. Tables 4 and 6 present the findings of the primary and secondary outcomes at the primary end point (6-months post-intervention).

**Table 5:** Summary of mixed model analysis of primary and secondary outcomes (changes from baseline): covariate adjusted analysis

Primary and secondary outcomes	Descriptive statistics				Mixed model analysis	
	Intervention arm		ETAU arm		Difference in mean (95% CI)	*p*-value
	*n*	*Mean (SD)*	*n*	*Mean (SD)*		
DD-CDAS—total disability score	221	− 7.34 (17.07)	239	− 4.50 (15.95)	− 2.74 (− 6.63,1.14)	0.1662
DD-CDAS—understanding and communication	221	− 9.16 (23.72)	239	− 7.79 (21.48)	− 2.68 (− 7.55, 2.19)	0.2797
DD-CDAS—getting around	221	− 5.63 (23.63)	239	− 2.91 (22.43)	− 3.01 (− 7.05, 1.03)	0.1442
DD-CDAS—self-care	221	− 10.29 (21.96)	239	− 7.03 (21.50)	− 1.50 (− 5.68, 2.69)	0.4826
DD-CDAS—getting along with people	221	− 7.04 (29.09)	239	− 2.91 (28.72)	− 4.33 (− 9.90, 1.23)	0.1268
DD-CDAS—participation in society	221	− 3.83 (25.35)	239	− 0.45 (24.44)	− 1.47 (− 7.10, 4.16)	0.6076
DD-CDAS—life activities	221	− 8.08 (24.30)	239	− 5.92 (23.01)	− 2.06 (− 6.83,2.71)	0.3965
PedsQL total score	221	7.17 (21.85)	237	0.77 (20.96)	5.35 (0.62, 10.09)	0.0268
PedsQL—parent health related Quality of life	221	7.08 (24.72)	237	1.90 (22.97)	4.70 (− 0.19, 9.58)	0.0597
PedsQL—family functioning	221	1.54 (27.29)	237	− 4.23 (25.93)	3.90 (− 1.63, 9.43)	0.1663
PedsQL—physical functioning	221	12.50 (31.14)	237	5.13 (28.64)	6.21 (− 0.10, 12.52)	0.0538
PedsQL—Emotional functioning	221	10.29 (29.08)	237	3.50 (30.44)	6.43 (0.82, 12.05)	0.0248
PedsQL—social functioning	221	1.67 (33.69)	237	− 1.50 (33.50)	3.63 (− 2.71, 9.98)	0.2605
PedsQL—cognitive functioning	221	1.72 (33.63)	237	− 0.84 (30.43)	1.41 (− 3.84, 6.66)	0.5967
PedsQL—communication	221	4.07 (30.07)	237	− 5.50 (32.18)	5.92 (− 0.09, 11.93)	0.0536
PedsQL—worry	221	18.43 (31.15)	237	7.99 (31.91)	6.76 (0.31, 13.21)	0.0401
PedsQL—daily activities	221	3.81 (36.08)	237	− 0.74 (39.50)	3.29 (− 4.89, 11.47)	0.4298
PedsQL—family relationship	221	0.18 (29.73)	237	− 6.31 (28.22)	3.99 (− 1.77, 9.75)	0.1744
SDQ total socio emotional difficulties	221	− 0.50 (6.54)	238	0.44 (6.69)	− 0.84 (− 2.25, 0.57)	0.2431
SDQ—emotional symptoms	221	− 0.17 (2.99)	238	0.11 (2.97)	− 0.40 (− 1.00, 0.19)	0.1863
SDQ—conduct problems	221	0.15 (2.12)	238	0.36 (2.21)	− 0.13 (− 0.56, 0.30)	0.5395
SDQ—hyperactivity symptoms	221	− 0.49 (2.74)	238	− 0.40 (2.61)	− 0.14 (− 0.55, 0.26)	0.4853
SDQ—peer problems	221	0.00 (2.34)	238	0.37 (2.65)	− 0.17 (− 0.68, 0.33)	0.4934
SDQ—pro social	221	0.31 (2.82)	238	0.27 (2.80)	− 0.11 (− 0.59, 0.36)	0.6401
FES total scores	221	1.41 (13.03)	237	1.18 (11.69)	0.41 (− 1.74, 2.57)	0.7085
FES-service system	221	5.50 (14.56)	237	4.79 (13.48)	0.47 (− 2.71, 3.65)	0.7711
FES—community involvement	221	193.00 (86.34)	237	180.97 (85.25)	13.09 (− 4.07, 30.25)	0.1346
FES—attitude	221	1.87 (10.22)	237	1.50 (9.81)	0.48 (− 1.04, 2.01)	0.5351
FES—knowledge	221	4.22 (11.47)	237	3.19 (11.21)	0.76 (− 1.69, 3.21)	0.5426
FES—behaviour	221	2.23 (11.14)	237	1.95 (10.32)	0.39 (− 1.78, 2.55)	0.7258
ISE	221	− 0.33 (2.41)	237	− 0.04 (2.56)	− 0.20 (− 0.69, 0.29)	0.4169

Adjusted for age, gender, and baseline DD-CDAS and DD-CGAS score

*ETAU* enhanced treatment-as-usual, *DD-CDAS* Developmental Disorders-Children Disability Assessment Schedule, *PedsQL* Paediatric Quality of Life Inventor—family impact module, *SDQ* Strengths and Difficulties Questionnaire, *FES* Family Assessment Schedule, *ISE* Inventory of Stigmatizing Experiences

**Table 6:** Summary of mixed model analysis of caregivers’ child interaction change from baseline

Caregivers’ child interaction	Domains of caregivers–chid interaction	Visit	Descriptive statistics				Mixed model analysis	
			Intervention arm		ETAU arm		Difference in mean (95% CI)	*p*-value
			*n*	*M (SD)*	*n*	*M (SD)*		
Play based activity	Child unengaged	Baseline	43	2.40 (1.73)	27	3.22 (2.06)		
		6 months post-intervention	61	2.43 (1.78)	47	2.77 (2.07)	0.77 (− 0.04, 1.58)	0.0614
	Child object engaged	Baseline	43	2.23 (1)	27	2.30 (1.14)		
		6 months post-intervention	61	2.51 (1.39)	47	3.04 (1.67)	− 0.04 (− 0.84, 0.76)	0.9103
	Child’s joint engagement	Baseline	43	3.67 (1.27)	27	3.07 (1.38)		
		6 months post-intervention	61	3.92 (1.56)	47	3.17 (1.51)	0.14 (− 0.76, 1.05)	0.7477
	Child’s stereotyped, restricted, repetitive behaviours	Baseline	43	1.53 (1.08)	27	2.74 (2.19)		
		6 months post-intervention	61	1.89 (1.55)	47	2.51 (1.98)	− 0.82 (− 2.02, 0.37)	0.1718
	Child’s attention to caregiver	Baseline	43	3.49 (1.30)	27	3.15 (1.41)		
		6 months post-intervention	61	3.80 (1.49)	47	3.64 (1.44)	− 0.10 (− 0.94, 0.74)	0.8100
	Child’s initiation of communication	Baseline	43	2 (1.18)	27	1.93 (1.21)		
		6 months post-intervention	61	2.25 (1.50)	47	1.62 (1.03)	0.21 (− 0.59, 1.01)	0.6021
	Child’s expressive language level and use	Baseline	43	2.30 (1.73)	27	2.19 (1.80)		
		6 months post-intervention	61	2.69 (2.09)	47	2.87 (2.05)	0.05 (− 0.67, 0.76)	0.8923
	Caregiver’s scaffolding	Baseline	43	2.74 (1.51)	27	2.1 (1.31)		
		6 months post-intervention	61	3.11 (1.90)	47	2.40 (1.68)	0.34 (− 0.69, 1.36)	0.5117
	Caregiver following in on child’s focus	Baseline	43	2.74 (1.35)	27	2.26 (1.40)		
		6 months post-intervention	61	3.23 (2.09)	47	2.57 (1.64)	− 0.04 (− 1.13, 1.06)	0.9480
	Caregiver’s affect	Baseline	43	2.93 (1.39)	27	2.48 (1.58)		
		6 months post-intervention	61	3.34 (1.97)	47	2.81 (1.56)	0.54 (− 0.24, 1.32)	0.1665
	Fluency and connectedness	Baseline	43	3.05 (1.40)	27	2.26 (1.23)		
		6 months post-intervention	61	3.31 (1.58)	47	2.94 (1.51)	0.18 (− 0.68, 1.03)	0.6801
	Shared routines and rituals	Baseline	43	2.42 (1.33)	27	1.85 (1.20)		
		6 months post-intervention	61	2.95 (1.83)	47	2.38 (1.61)	− 0.01 (− 1.04, 1.01)	0.9827
Home routine	Child unengaged	Baseline	39	2.28 (2.06)	23	4.35 (2.48)		
		6 months post-intervention	37	1.92 (1.72)	23	4 (2.32)	− 0.21 (− 1.82, 1.39)	0.7821
	Child object engaged	Baseline	39	1.56 (1.14)	23	1.48 (0.79)		
		6 months post-intervention	37	1.81 (1.22)	23	1.91 (1.08)	0.11 (− 0.88, 1.10)	0.8124
	Child’s joint engagement	Baseline	39	4.03 (2.08)	23	2.87 (1.46)		
		6 months post-intervention	37	3.86 (1.40)	23	3.17 (1.40)	0.31 (− 0.97, 1.59)	0.6211
	Child’s stereotyped, restricted, repetitive behaviours	Baseline	39	1.87 (1.38)	23	2.04 (1.58)		
		6 months post-intervention	37	1.27 (0.65)	23	1.65 (1.23)	− 0.52 (− 1.54, 0.50)	0.3000
	Child’s attention to caregiver	Baseline	39	3.69 (1.52)	23	3.52 (1.34)		
		6 months post-intervention	37	4.27 (1.45)	23	3.09 (1.12)	0.67 (− 0.69, 2.03)	0.3135
	Child’s initiation of communication	Baseline	39	1.69 (0.83)	23	1.39 (0.58)		
		6 months post-intervention	37	2 (1)	23	1.57 (0.90)	− 0.24 (− 0.96, 0.48)	0.4887
	Child’s expressive language level and use	Baseline	39	2.26 (1.62)	23	2.93 (1.850)		
		6 months post-intervention	37	3.27 (1.92)	23	3.65 (1.82)	− 0.19 (− 1.47, 1.09)	0.7621
	Caregiver’s scaffolding	Baseline	39	2.82 (1.97)	23	2.26 (1.54)		
		6 months post-intervention	37	3.14 (1.78)	23	1.83 (1.15)	1.20 (− 0.24, 2.64)	0.0981
	Caregiver following in on child’s focus	Baseline	39	2.77 (2.15)	23	2 (1.45)		
		6 months post-intervention	37	3.35 (1.83)	23	1.65 (1.19)	0.95 (− 0.59, 2.49)	0.2117
	Caregiver’s affect	Baseline	39	2.77 (1.68)	23	2.35 (1.34)		
		6 months post-intervention	37	3.30 (1.63)	23	1.87 (1.22)	1.04 (− 0.38, 2.46)	0.1404
	Fluency and connectedness	Baseline	39	2.87 (1.61)	23	2.30 (1.18)		
		6 months post-intervention	37	3.57 (1.28)	23	2.65 (1.40)	0.79 (− 0.24, 1.83)	0.1244
	Shared routines and rituals	Baseline	39	3.13 (1.88)	23	2.52 (1.50)		
		6 months post-intervention	37	3.51 (1.50)	23	2.65 (1.72)	0.69 (− 0.49, 1.88)	0.2360

All Family Volunteers (FVs) were females (95% mothers and 5% paternal aunts) with the mean age of 39 years (± 4.38). Out of 62 potential FVs trained by the trainers, 36 delivered the program to caregivers in local villages. Remaining 26 FVs were not able to deliver the program; due to health problems (n = 7); competing family commitments (n = 7); they were working mothers (n = 6) and did not achieve competency (n = 6). A total of 504 group sessions were delivered by 36 FVs in 79 village-based groups. The average group size was 6 (range 5 to 7). Out of 270, 230 (85%) of the trial participants attended 6 sessions (± 1). Average duration of a group session was 1 h 42 min. FVs organized group supervision sessions with 178 caregivers after 9-training sessions. The attendance in these sessions was 73%.

We assessed 20% (103/504) of sessions delivered by the 36 FVs using ENACT to evaluate program fidelity. The intervention sessions were delivered with good fidelity as all FVs scored 2.5 or more (mean [SD], 2.97 ± 0.21) on all items of adapted ENACT. Over the 6-months duration of program delivery, FVs were supervised in 30 group supervision meetings by the trainers. The attendance in the group supervision sessions was 75%.

All caregivers were provided feedback about the assessment result of their child and informed about the options for seeking appropriate care. During the 6-month follow-up period, 80.5% (178/270) participants in intervention arm and 78% (187/237) of caregivers in the ETAU arm contacted a primary health care physician for their child’s health related issues. Medicines including vitamins, calcium and iron supplements were prescribed to 88.2% (195/270) participants in intervention arm and 83.3% (199/270) participants in control arm. The details on health services utilization are mentioned in Table 2.

The allocation status of only 1 cluster (13%) in the intervention arm and 2 clusters (20%) in ETAU arm was correctly guessed by outcome assessors after the primary outcome assessment at post-intervention, indicating that blinding was successful.

## Discussion

The results of our study show that a technology-assisted, family-volunteer delivered, brief, multicomponent, parents’ skills training did not improve child functioning beyond those in the ETAU arm. Our sample consisted of heterogeneous group of children with a range of developmental conditions such as intellectual disability, motor difficulties, speech and communication difficulties and Down syndrome. Although in statistical analysis, child’s age, gender and baseline severity did not impact the effect of intervention, the heterogeneity of the study sample might have led to inadequate power to evaluate a meaningful difference in each sub-group of the current study sample. Another possible explanation of lack of improvement in child’s functioning could be a relatively short intervention period that was inadequate to bring a clinically meaningful change in child’s functional disability and other socioemotional outcomes. Finally, we used a cluster randomised trial design to minimise the risk of contamination; however, given that the intervention delivery agents in the study were family volunteers working in closely knit rural community settings of Pakistan, the risk of contamination between the study arms cannot be ruled-out. The intervention led to significant improvement in caregivers’ quality of life. These results are consistent with other studies reported in the literature, where, family focused, parent-mediated interventions improved parental and family functioning [7, 30]. These findings become more significant in the context of South Asia, where mostly the mothers carry the main burden of caring for children with developmental disorders and while doing so they experience high emotional burden and stress [31–35]. Our village based ‘family networks’ intervention model provides an opportunity to the caregivers to discuss their challenges in taking care of a child with developmental disorder with their peers/family volunteers in a non-stigmatizing and supportive environment. This may have resulted in improved emotional wellbeing and reduced worries of caregivers associated with taking care of a child with developmental disorder. Parental health related quality of life is an important proximal outcome of a parent mediated intervention and it may be related to positive developmental outcomes in children with developmental delays in later years.

In the present study, we used technology to enhance our previous work on parent-mediated interventions, implemented in two of the most populous South Asian countries [36]. We enhanced our intervention delivery by developing android application hosted on a tablet device to train caregivers and taking task-shifting to its proximal level by training Family Volunteers (FVs) to deliver intervention to caregivers of children with developmental in a rural Pakistan. The results of the program fidelity demonstrated the use of technology as an effective innovation to train caregivers in evidence-based psychosocial interventions in low resource settings. Digital technology in any form (web based platforms, discussion forums, mobile devices) has a potential to deliver parent-mediated interventions [37], especially to scale-up interventions in low resource settings [38] and minimize the risk of reduction in program fidelity and dosage of intervention delivery by non-specialists, which are the frequent concerns associated with the task-shifting in global mental health [39].

Existing child mental health approaches rely on weak and fragmented health or social care systems that are incapable of providing evidence-based services to children with developmental disorders at-scale and sustainably in low resource settings. Our model of service delivery provides its own motivated human-resource and culturally adapted technology-assisted training platform. These technological and social innovations can work in synergy with the existing health care systems to provide an integrated and innovative [40] system of care that can be sustained with minimal support from local governmental and non-governmental agencies. Such innovative, community-based models of service delivery can serve as a starting point of a cost-effective and potentially sustainable stepped-care model of service delivery for childhood developmental disorders in low resource settings globally [41, 42].

Ever since its launch, the WHO mhGAP-IG has created a huge impact globally. It is being implemented in more than 90 countries of the world to bridge the service gap, with the ultimate aim to achieve the Universal Health Coverage for mental health [43]. Despite its huge uptake, globally, the impact evaluation of WHO mhGAP-IG has only been restricted to small studies, with less information on real-world contextual challenges and scientific learnings [44–46]. Our study is the first substantive randomised controlled trial of a non-specialists’ delivered WHO mhGAP-IG based caregivers’ skills training intervention for management of children with developmental disorders in low resource community settings. Although, our study did not result in improving child outcomes at 6-months post-intervention, the lessons learned from the present study have methodological implications on scaling-up care for children with developmental disorders in low resource settings globally. We recruited participants following the trans-diagnostic approach of WHO mhGAP program, which resulted in a heterogeneous group of children with a wide age range and a range of developmental conditions such as intellectual disability, motor difficulties, speech and communication difficulties and Down syndrome. Future studies may benefit from using adaptive interventions designs [47, 48] to cater the specific developmental needs of diverse group of children with developmental disorders.

There are several limitations in our study. Although, the caregivers’ skills training resulted in improved parental health related quality of life, the results of evaluation did not demonstrate improvement in child outcomes and we are unable to comment if improved parental health related quality of life translated in enhancing caregivers’ competency to interact and engage with the child due to high refusal rate from the community to video record mother–child interaction. Another limitation was brief duration of intervention delivery (6 months), given the diversity of conditions included in the category of developmental disorders; different degree of severity of symptoms of developmental disorders and delays in the target group, and wide age range of the study sample. These limitations call for pragmatic outcome measures [49], adaptive interventions [47, 48] and Sequential Multiple Assignment Randomized Trial (SMART) trial designs [50] to evaluate the impact of a caregivers skills training intervention for children with developmental disorders in real-world settings.

## Conclusions

The treatment gap for community-based interventions for children with developmental disorders in low resource settings globally remains nearly 100% and research to address barriers to scaling-up care for childhood developmental disorders remain a neglected area. Innovations such as training caregivers and lay health workers using technology may address such bottlenecks to bridge the treatment gap for developmental disorders in low resource settings. WHO mhGAP-IG based caregivers’ skills training is a potentially scalable intervention; however, methodological innovations such as pragmatic outcome measures and SMART trials are needed to evaluate the impact in real-world settings.

## Data Availability

The dataset generated and analysed during the current study are available from the corresponding author on a reasonable request.

## Abbreviations

WHO mhGAP-IG: World Health Organization’s mental health Gap-Intervention (mhGAP-IG)
ETAU: Enhanced treatment-as-usual
DD-CDAS: Childhood Disability Assessment Schedule for Developmental Disorders
AMD: Adjusted Mean Difference
ASD: Autism spectrum disorder
ID: Intellectual disabilities
FVs: Family volunteers
cRCT: Cluster Randomized Controlled Trial
UCs: Union Councils
ADHD: Attention Deficit Hyperactivity Disorder
ENACT: ENhancing Assessment of Common Therapeutic factors
LHWs: Lady Health Workers
WHODAS: WHO Disability Assessment Schedule
PedsQL: Paediatric Quality of Life
CCI: Caregiver–child interaction
SDQ: Strengths and Difficulties Questionnaire
FES: Family Empowerment Scale
ISE: Inventory of Stigmatizing Experiences
ICC: Intra-cluster correlation coefficient
TQS: Ten Questions Screening
CONSORT: Consolidated Standards of Reporting Trials
ICF-CY: International Classification of Functioning, Disability and Health-Child and Youth
